# Impact of chronic kidney disease on long-term outcome of patients with valvular heart defects

**DOI:** 10.1007/s11255-020-02561-4

**Published:** 2020-07-14

**Authors:** Łukasz Kuźma, Jolanta Małyszko, Hanna Bachórzewska-Gajewska, Marta Maria Niwińska, Anna Kurasz, Małgorzata Zalewska-Adamiec, Marcin Kożuch, Sławomir Dobrzycki

**Affiliations:** 1 Department of Invasive Cardiology, Medical University of Bialystok, Bialystok, Poland; 2 Department of Nephrology, Dialysis and Internal Medicine, Medical University of Warsaw, ul. Banacha 1a, 02-097 Warsaw, Poland; 3 Department of Clinical Medicine, Medical University of Bialystok, Bialystok, Poland

**Keywords:** Chronic kidney disease, Glomerular filtration rate, Valvular heart disease, Aortic stenosis, Mitral insufficiency

## Abstract

**Introduction:**

Valvular heart diseases (VHD) are becoming a significant problem in the Polish population. Coexistence of chronic kidney disease (CKD) in patients with VHD increases the risk of death and affects further therapeutic strategy.

**Aim:**

Analysis impact of CKD on long-term prognosis in patients with VHD.

**Material and methods:**

The inclusion criteria were met by 1025 patients with moderate and severe VHD. Mean observation time was 2528 ± 1454 days.

**Results:**

The average age of the studied population was 66.75 (SD = 10.34), male gender was dominant 56% (*N* = 579). Severe aortic valve stenosis (AVS) occurred in 28.2%, severe mitral valve insufficiency (MVI) in 20%. CKD occurred in 37.1% (*N* = 380) patients mostly with mitral stenosis (50%, *N* = 16) and those with severe MVI (44.8%, *N* = 94). During the observational period, 52.7% (*N* = 540) deaths were noted. Increased risk of mortality was associated mostly with age (OR: 1.02, 95% CI: 1.00–1.03, *p* < 0.001), creatinine (OR:1.27, 95% CI: 1.12–1.43, *p* < 0.001), CKD (OR: 1.30, 95% CI: 1.17–1.44, *p* < 0.001), reduced ejection fraction (EF) (OR: 0.98, 95% CI: 0.97–0.99, *p* = 0.01) and coexisting of AVS (OR: 1.19, 95% CI: 1.04–1.35, *p* = 0.01).

**Conclusions:**

Mitral valve defects more often than aortic valve defects coexist with chronic kidney disease. Regardless of the stage, chronic kidney disease is an additional factor affecting the prognosis in patients with heart defects. Factors increasing the risk of death were age, creatinine concentration and reduced EF. The monitoring of renal function in patients with VHD should be crucial as well as the implementation of treatment at an early stage.

## Introduction

Cardiovascular diseases are the leading cause of mortality among the Polish population according to the data from WHO from 2018. These are responsible for 46% of deaths—it is almost twice as much as deaths caused by cancer (27%) [1].

In the case of cardiovascular diseases, the subject of heart valve defects is increasingly more common. Over time, with the development of medicine and the spread of antibiotics, degenerative valve disease has become the most common form of valvular defect outgrowing rheumatic cause [2]. Currently, there are guidelines essential for the management of valvular defects—this problem is growing continuously, with more and more complications. The Gold Standard treatment for heart valve diseases is percutaneous or surgical valve intervention for those patients who meet certain criteria [3–5]. In those cases, invasive intervention improves not only the comfort of patients’ lives, but also the prognosis [4, 6].

The growing population of people with valvular defects and chronic kidney disease (CKD) and also the lack of new research on this issue makes it necessary to explore this matter. It seems that in the face of medical progress and improvement of minimally invasive methods of treatment of valvular defects, having up-to-date data on the subject, number and severity of defects is extremely important.

It is a well-known fact that coexisting disorders have a negative impact on the heart condition. Kidney dysfunction induces chronic or acute heart dysfunction. This association between the destructive impacts of both of the above-mentioned organs on each other is in the medical nomenclature named “cardiorenal syndrome”. Predominantly, it affects cardiac function, especially the function of the left ventricle [7, 8]. Lots of authors claim that any glomerular filtration rate (GFR) lowering and chronic kidney disease affects directly not only the myocardium, but also the condition of heart valves. Impaired kidney function contributes to vascular damage, increasing the concentration of inflammatory mediators and factors predisposing to the formation of calcifications with a simultaneous decrease in the concentration of calcination inhibitors (impaired calcium-phosphate balance). This leads to the calcination of vessel walls and also heart valves [9, 10].

Therefore, we aimed to compare the occurrence of left-sided heart valve diseases in the population with and without CKD and to observe which comorbidities and factors have an impact on patients’ mortality.

## Patients and methods

Medical records of 12,954 patients hospitalized in the Department of Invasive Cardiology of the Medical University of Bialystok in the period 2006–2010 were analyzed.

The assignment of patients to particular groups was based on the ESC guidelines for the management of valvular heart disease published in 2017 [3]. Prior cardiosurgical valve replacement was the exclusion criterion.

1025 patients with valvular heart disease in moderate and severe stages met the inclusion criteria. The set of variables subjected to interpretation consisted of demographic data, medical history, coronary angiogram, percutaneous and surgical treatment.

Estimated glomerular filtration rate was counted using three formulas: MDRD, Cockcroft-Gault, CKD-EPI. Chronic kidney disease is defined as the presence of kidney damage or an estimated glomerular filtration rate (eGFR) less than 60 ml/min/1.73 m^2^, persisting for 3 months or more, irrespective of the cause. CKD recognition was made according to KDIGO 2012 Clinical Practice Guideline for the Evaluation and Management of Chronic Kidney Disease [11].

A follow-up study included the analysis of medical documentation of the Cardiosurgery Department and the Invasive Cardiology Department of the Medical University of Bialystok, phone call interview and provided information on the duration and type of procedure performed. There was no follow-up concerning the aforementioned data in 26 (2.5%) cases.

All-cause mortality was collected from the Polish Ministry of Digital Affairs.

The mean follow-up period from the onset to the procedure was 360 ± 796 days and then from the cardiosurgical procedure to death it was 2480 ± 1445 days. The complete follow-up duration was 2528 ± 1454 days.

The study was approved by the Local Bioethics Committee of the Medical University of Bialystok no. R-I-002/20/2013.

### Statistical analysis

In statistical analysis, the distribution of variables was evaluated using the Kolmogorov–Smirnov test. Two-tailed *T*-test and ANOVA test were used for comparative analysis. Non‐normally distributed data were compared with the Mann–Whitney and Kruskala–Wallis tests. For multiple pairwise comparisons the Steel—Dwass–Critchlow–Fligner two-tailed test was used. Obtained results were presented as mean values with standard deviation or percentage values corresponding to relative frequency.

Spearman’s rank correlation test was applied for evaluating the relationships between eGFR and mortality among patients with and without CKD. Multivariable logistic regression backward stepwise Wald method was used to determine mortality risk factors. Death at long-term observation was used as the explanatory variable, as the variables explaining we used the clinical data of the patient on admission and the variables obtained during the follow-up period. To reduce the number of predictors in each step after *F*-test a non-significant variable was remain. In final the significant results were presented as risk ratio (RR) from the 5th to the 95th.

The threshold of statistical significance was set at *P* < 0.05 for all the tests. All analyses were performed using MS Excel and SPSS IBM Software.

## Results

**Fig. 1:**
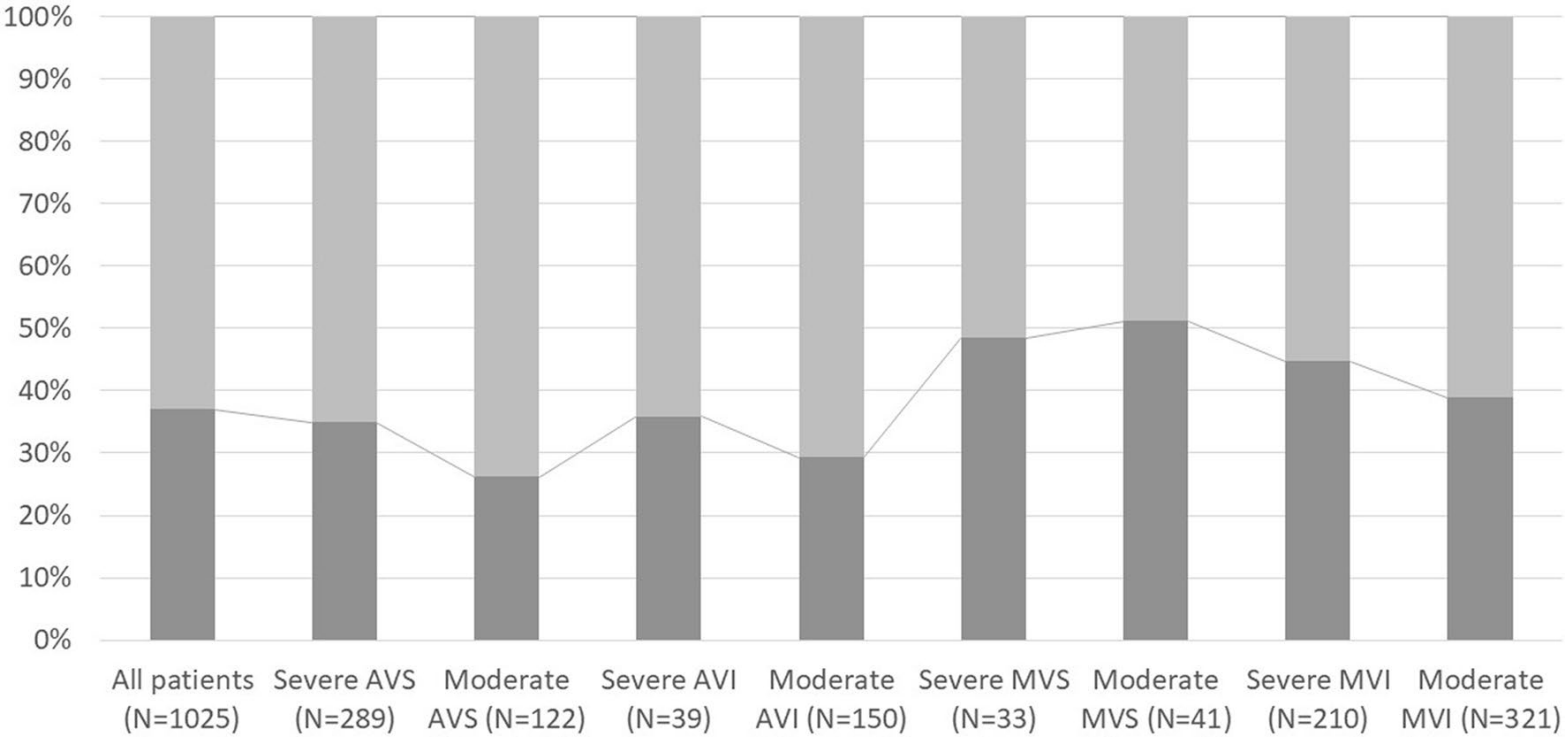
Chronic kidney disease among patients with valvular heart defect

In the studied population, CKD occurred in 37.1% (*N* = 380) patients. CKD affected mostly patients with mitral stenosis (50%, *N* = 16) and those with severe mitral insufficiency (44.8%, *N* = 94). Among the group of patients with severe mitral regurgitation, the percentage of patients with CKD was greater than in other groups [44.8% (*N* = 94) vs. 35.1% (*N* = 286), *p* = 0.010]. The lowest incidence of CKD was observed in the group of patients with moderate aortic stenosis [26.2% (*N* = 32) vs. 38.5% (*N* = 348), *p* = 0.008], [29.3%, *N* = 44 vs. 38.4%, *N* = 336, *p* = 0.034]. Among the group of patients with severe heart valve defects, CKD affected 40.4% (*N* = 238) of the population, while in the group with moderate heart valve defects it was 32.6% (*N* = 142) [Fig. 1].

The greatest percentage of patients were characterized by eGFR in the range 60–90 ml/min/1.73 m^2^ 51.5% (*N* = 528). The next largest group in terms of quantity were patients with eGFR in the range of 45–60 ml/min/1.73 m^2^, that is 22.5% (*N* = 231). There have been single cases of patients with end-stage CKD 0.8% (*N* = 8), seven of whom were on dialysis (*N* = 7) [Table 1].

**Table 1:** The occurrence of valvular defects in the study population according to the eGFR value

eGFR CKD -EPI (ml/min/1.73 m^2^)	eGFR > 90	eGFR 60—90	eGFR 45–60	eGFR 30—45	eGFR 15—30	eGFR < 15	Kruskal–Wallis test
All patients (*N* = 1025)	**13.8%** **(N = 141)**	**51.5%** **(N = 528)**	**22.5%** **(N = 231)**	**8.4%** **(N = 86)**	**3%** **(N = 31)**	**0.8%** **(N = 8)**	*p* < 0.001
Moderate VHD (*N* = 436)	**14.7%** **(n = 62)**	**54.3%** **(n = 237)**	**19.8%** **(n = 87)**	**7.8%** **(n = 34)**	**2.52%** **(n = 11)**	**0.7%** **(n = 3)**	*p* < 0.001
Severe VHD (*N* = 589)	**13.1%** **(n = 77)**	**49.4%** **(n = 291)**	**24.5%** **(n = 144)**	8.82% (*n* = 52)	**3.9%** **(n = 20)**	**0.8%** **(n = 5)**	*p* < 0.001
Severe AVS (*N* = 289)	**11.8%** **(n = 34)**	**55.4%** **(n = 160)**	**21.5%** **(n = 62)**	7.3% (*n* = 21)	**3.1%** **(n = 9)**	**1%** **(n = 3)**	*p* < 0.001
Moderate AVS (*N* = 122)	**10.7%** **(n = 13)**	**63.9%** **(n = 78)**	18% (*n* = 22)	4.9% (*n* = 6)	2.5% (*n* = 3)	0% (*n* = 0)	*p* < 0.001
Severe AVI (*N* = 39)	**25.6%** **(n = 10)**	46.2% (*n* = 18)	20.5% (*n* = 8)	7.7% (*n* = 3)	0% (*n* = 0)	0% (*n* = 0)	*p* < 0.001
Moderate AVI (*N* = 150)	16.7% (*n* = 25)	**56%** **(n = 84)**	20% (*n* = 30)	**6%** **(n = 9)**	**1.3%** **(n = 2)**	0% (*n* = 0)	*p* < 0.001
Severe MVS (*N* = 33)	**9.1%** **(n = 3)**	**45.5%** **(n = 15)**	27.3% (*n* = 9)	9.1% (*n* = 3)	6.1% (*n* = 2)	3% (*n* = 1)	*p* < 0.001
Moderate MVS (*N* = 41)	**7.3%** **(n = 3)**	**43.9%** **(n = 18)**	**41.5%** **(n = 17)**	4.9% (*n* = 2)	2.4% (*n* = 1)	0% (*n* = 0)	*p* < 0.001
Severe MVI (*N* = 210)	**12.4%** **(n = 26)**	**46.2%** **(n = 97)**	**26.2%** **(n = 55)**	11.4% (*n* = 24)	**3.8%** **(n = 8)**	0% (*n* = 0**)**	*p* < 0.001
Moderate MVI (*N* = 321)	**13.4%** **(n = 43)**	**49.8%** **(n = 160)**	**22.1%** **(n = 71)**	10.3% (*n* = 33)	**2.5%** **(n = 8)**	**1.9%** **(n = 6)**	*p* < 0.001

Significant differences between groups using the Steel–Dwass–Critchlow–Fligner are highlighted, *p*  < 0.05

*AVI* aortic valve insufficiency, *AVS* aortic valve stenosis, *MVI* mitral valve insufficiency, *MVS* mitral valve stenosis, *N* number of patients, *n* number of valvular defects

In the analysis of a group of patients with proper kidney function (eGFR > 90 ml/min/1.73 m^2^), we found a greater percentage of patients with severe aortic insufficiency, and it was higher than in other groups (25.6% *N* = 10 vs. 13.2% *N* = 131, *p* = 0.028). In the subgroup with eGFR 60–90 ml/min/1.73 m^2^,the most common valvular defect was moderate aortic stenosis and it was greater than in other groups (63.9% *N* = 78 vs. 48.8% *N* = 368, *p* = 0.003). Moderate eGFR lowering (30–60 ml/min/1.73 m^2^) was mostly observed among patients with mitral stenosis (46.4%, *N* = 19) and mitral insufficiency 37.6% (*N* = 79) in the severe and moderate stage. In the case of patients with moderate mitral stenosis, CKD in the G3a stage occurred significantly more often than in other groups (41.5% *N* = 17 vs. 21.7% *N* = 214, *p* = 0.006). Occurrence of severe eGFR lowering was mostly related to patients with moderate and severe mitral insufficiency (4.1%, *N* = 22). Furthermore, end-stage CKD occurred in eight patients, in which 75% of them had mitral insufficiency.

The average age of the studied population was 66.75 (SD = 10.3) and male gender was dominant 56% (*N* = 579). Population of patients with chronic kidney disease was significantly older (71.5 SD = 9.1 vs. 64.0 SD = 10.0, *p* < 0.001) and characterized by lower percentage of males (47% *N* = 178 vs. 62% *N* = 401, *p* < 0.001). Most common coexisting diseases were: coronary artery disease (CAD) 69% (*N* = 708), arterial hypertension (HA) 61% (*N* = 625) and atrial fibrillation (AF) 39% (*N* = 400). Presence of these comorbidities was greater in population with CKD [78% (*N* = 295) vs. 64% (*N* = 413), *p* < 0.001], [65% (*N* = 248) vs. 58% (*N* = 377), *p* = 0.031], [49% (*N* = 186) vs. 33% (*N* = 214), *p* < 0.001] (Table 2).

**Table 2:** Comparison between patients with and without chronic kidney disease

	All patients (*N* = 1025)	CKD (*N* = 380)	Non-CKD (*N* = 645)	*p*
Male, % (*N*)	56 (579)	47 (178)	62 (401)	*p* < 0.001
Age, (years), mean (SD)	66.75 (10.3)	71.5 (9.1)	64.0 (10.0)	*p* < 0.001
BMI, (kg/m^2^), mean (SD)	27.59 (4.61)	27.3 (4.4)	27.8 (4.7)	*p* = 0.190
CCS, mean (SD)	0.96 (1.12)	1.2 (1.2)	0.8 (1.1)	*p* < 0.001
NYHA, mean (SD)	2.31 (0.62)	2.4 (0.7)	2.2 (0.6)	*p* < 0.001
HA, % (*N*)	61 (625)	65 (248)	58 (377)	*p* = 0.031
HL, % (*N*)	35 (360)	31 (118)	38 (242)	*p* = 0.036
DM, % (*N*)	22 (229)	27 (102)	20 (127)	*p* = 0.008
HU, % (*N*)	3 (35)	5 (20)	2 (15)	*p* = 0.012
AF, % (*N*)	39 (400)	49 (186)	33 (214)	*p* < 0.001
HFrEF, % (*N*)	26 (226)	30 (99)	23 (127)	*p* = 0.017
CAD, % (*N*)	69 708)	78 (295)	64 (413)	*p* < 0.001
SVD, % (*N*)	14 (147)	15 (58)	14 (89)	*p* = 0.518
MVD, % (*N*)	28 (287)	37 (142)	22 (145)	*p* < 0.001
ACS, % (*N*)	22 (223)	29 (112)	17 (111)	*p* < 0.001
Creatinine (mg/dl), mean, (SD)	1.11 (0.6)	1.4 (0.8)	0.9 (0.2)	*p* < 0.001
CKD-EPI eGFR ml/min/1.73 m^2^, % (*N*)	68.05 (20)	48.1 (14.2)	79.8 (12.1)	*p* < 0.001
Hemoglobin	13.41 (1.58)	13.0 (1.7)	13.7 (1.47)	*p* < 0.001
ALT (IU/l), median (IQR)	26 (13)	26 (10)	25 (8)	*p* = 0.028
LDL (mg/dl), mean (SD)	107.2 (38.4)	103.8 (42.1)	109.2 (35.9)	*p* = 0038
HDL (mg/dl), mean (SD)	46.1 (13.2)	44.4 (13.4)	47.0 (12.9)	*p* = 0.004
Cholesterol (mg/dl), mean (SD)	177.4 (44.9)	170.2 (45.8)	181.5 (43.8)	*p* < 0.001
Triglycerides (mg/dl), median (IQR)	102 (41.8)	101 (36.5)	102 (41.8)	*p* = 0.861
Fibrinogen (mg/dl), mean (SD)	412.7 (105.4)	433.8 (114.9)	400.3 (97.4)	*p* < 0.001
ACEI, % (*N*)	68 (696)	69 (261)	67 (435)	*p* = 0.385
ARB, % (*N*	8 (87)	9 (36)	8 (51)	*p* = 0.756
β-blockers, % (*N*)	82 (842)	83 (314)	82 (528)	*p* = 0.378
Statins, % (*N*)	59 (605)	61 (231)	58 (374)	*p* = 0.017
Loop diuretic, % (*N*)	49 (506)	62 (235)	42 (271)	*p* < 0.001
MCRA, % (*N*)	44 (453)	54 (205)	38 (248)	*p* < 0.001
Thiazide, % (*N*)	9 (94)	9 (33)	9 (61)	*p* = 0.679
Ao (mm), mean (SD)	37.0 (7.4)	3.9 (6.0)	37.7 (8.04)	*p* < 0.001
IVS (mm), mean (SD)	12.9 (2.7)	12.8 (2.8)	12.9 (2.6)	*p* = 0.574
ILW (mm), mean (SD)	11.9 (2.6)	11.8 (2.1)	12.0 (2.8)	*p* = 0.466
LA (mm), mean (SD)	45.8 (9.3)	47.1 (10.0)	45.1 (8.8)	*p* < 0.001
LV (mm), mean (SD)	54.3 (9.4)	54.1 (9.2)	54.4 (9.5)	*p* = 0.673
EF (%), mean (SD)	47.9 (13.8)	46.1 (14.4)	49.0 (13.2)	*p* = 0.003

*CCS* chronic coronary syndromes, *HA* arterial hypertension, *HL* hyperlipidemia, *DM* diabetes mellitus, *HU* hyperuricemia, *AF* atrial fibrillation, *HFrEF* heart failure with reduced ejection fraction, *CAD* coronary artery disease, *SVD* single vessel disease, *MVD* multi vessel disease, *ACS* acute coronary syndromes, *ALT* alanine aminotransferase, *LDL* low density lipoprotein, *HDL* high density lipoprotein, *ACEI* angiotensin converting enzyme inhibitor, *ARB* angiotensin receptor blocker, *MCRA* mineral corticosteroid receptor antagonist, *Ao* aorta, *IVS* interventricular septum, *ILW* inferolateral wall, *LV* left ventricle, *LA* left atrium, *EF* ejection fraction

The biochemical data analysis showed a decrease in total cholesterol [170.2 (SD = 45.8) vs. 181.48 (SD = 43.8), *p* < 0.001] and low-density lipoprotein (LDL) [103.8 (SD = 42.1) vs. 109.2 (SD = 35.9), *p* = 0.038] levels among patients with CKD. This group was also characterized by higher fibrinogen concentration [433.8 (SD = 114.9) vs. 400.3 (SD = 97.5), *p* < 0.001]. Loop diuretics (62% *N* = 235 vs. 42% *N* = 271, *p* < 0.001) and mineral corticosteroid receptor antagonist (MCRA) (54% *N* = 205 vs. 38% *N* = 248, *p* < 0.001) usage was greater in the group of patients with CKD. They also had a greater left atrium dimension (47.1 SD = 10.0 vs. 45.1 SD 8.8, *p* > 0.001) and lower ejection fraction (46.1 SD = 14.4 vs. 49.0 SD = 13.2, *p* < 0.001) (Table 2).

**Fig. 2:**
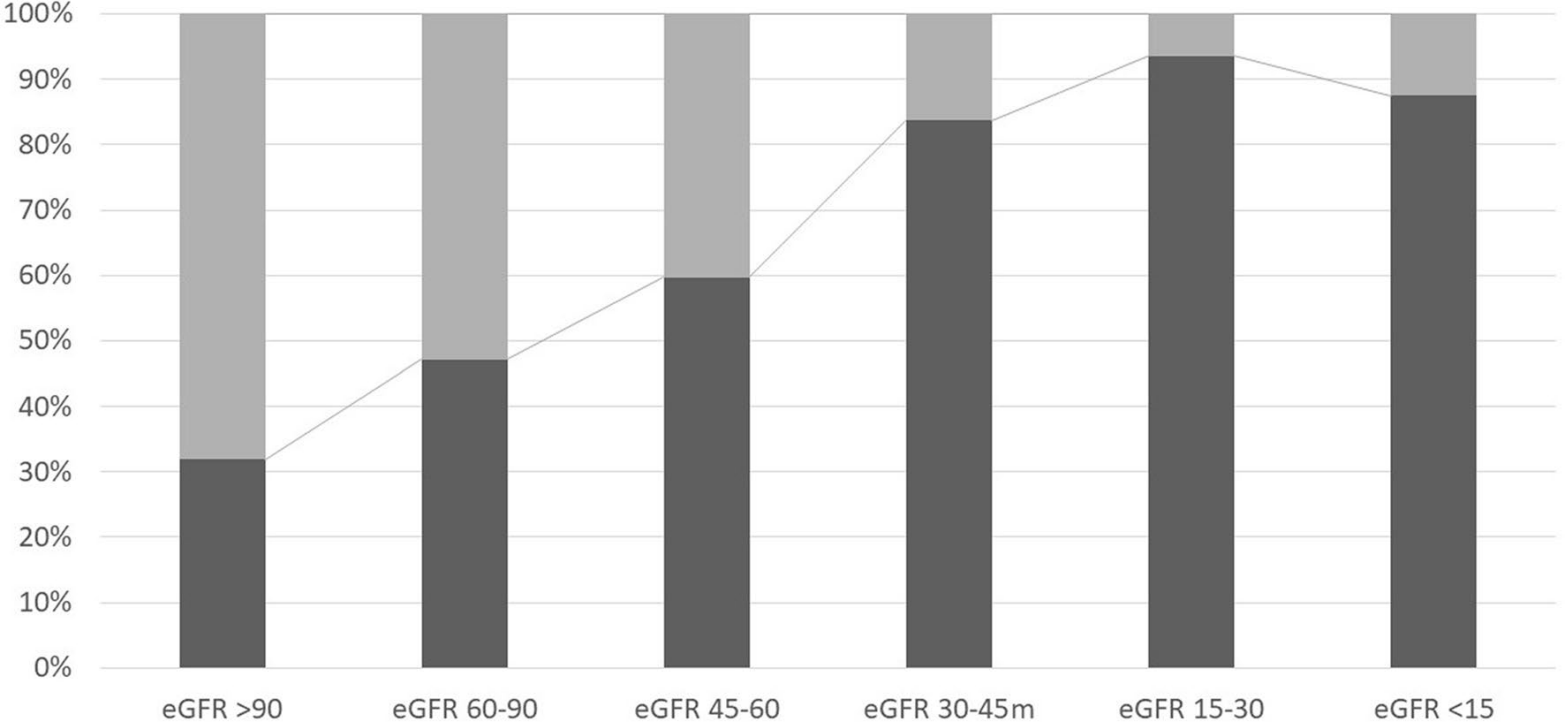
The percentage of deaths depending to the eGFR (Kruskal–Wallis test *p* < 0.001; multiple comparisons using the Steel–Dwass–Critchlow–Fligner *p*  < 0.001 for all groups)

During the observational period of whole population 52.7% (*N* = 540) deaths were noted. Coexisting diseases differed between living and deceased patients, except for arterial hypertension. Moreover, severe mitral insufficiency was the predominant cause of mortality 62.9% (*N* = 132). The greater percentage of deaths was noted in patients with end-stage CKD, that is 87.5% (*N* = 7) (Fig. 2). For coexisting diseases greater prevalence in the dead group was noted in the case of hyperuricemia 74.3% (*N* = 26), acute coronary syndromes 71.8% (*N* = 160) and coronary disease with significant atherosclerosis lesions 67.5% (*N* = 293) (Table 3).

**Table 3:** Comparison of alive and dead patients

	Dead patients 52.7% (*N* = 540)	Alive patients 47.3% (*N* = 485)	*p*
Male, % (*N*)	63.3 (10.0)	69.9 (9.6)	*p* < 0.001
Age (years), mean (SD)	53.9 (312)	46.1 (267)	*p* < 0.001
BMI, (kg/m^2^), mean (SD)	27.4 (4.9)	27.9 (2.4)	*p* < 0.001
CCS, mean (SD)	1.1 (1.1)	0.8 (1.1)	*p* = 0.015
NYHA, mean (SD)	2.4 (0.6)	2.2 (0.6)	*p* < 0.001
HA, % (*N*)	51.9 (325)	48.1 (300)	*p* = 0.321
HL, % (*N*)	62.9 (144)	37.1 (85)	*p* < 0.001
DM, % (*N*)	74.3 (26)	25.7 (9)	*p* = 0.009
HU, % (*N*)	52.7 (243)	47.3 (157)	*p* < 0.001
Severe AVS, % (*N*)	51.6 (149)	48.4 (140)	*p* = 0.651
Severe AVI, % (*N*)	30.1 (12)	69.2 (27)	*p* = 0.005
Severe MVS, % (*N*)	42.4 (14)	57.6 (19)	*p* = 0.223
Severe MVI, % (*N*)	62.9 (132)	37.1 (78)	*p* < 0.001
CKD, % (*N*)	69 (262)	31 (118)	*p* < 0.001
eGFR > 90 ml/min/1.73m^2^, % (*N*)	32 (45)	68 (96)	*p* < 0.001
eGFR 60–90 ml/min/1.73m^2^, % (*N*)	47.2 (249)	52.8 (*N* = 279)	*p* < 0.001
eGFR 45–60 ml/min/1.73m^2^, % (*N*)	59.8 (138)	40.2 (*N* = 93)	*p* = 0.015
eGFR 30–45 ml/min/1.73m^2^, % (*N*)	83.7 (72)	16.3 (*N* = 14)	*p* < 0.001
eGFR 15–30 ml/min/1.73m^2^, % (*N*)	93.5 (29)	6.5 (*N* = 2)	*p* < 0.001
eGFR < 15 ml/min/1.73m^2^, % (*N*)	87.5 (7)	12.5 (*N* = 1)	*p* = 0.103
CAD, % (*N*)	55.5 (417)	44.5 (*N* = 335)	*p* < 0.001
ACS, % (*N*)	71.8 (160)	28.2 (*N* = 63)	*p* < 0.001

*BMI* body mass index, *CCS* chronic coronary syndromes, *NYHA* New York Heart Association, *HA* arterial hypertension, *DM* diabetes mellitus, *HU* hyperuricemia, *AF* atrial fibrillation, *AVS* aortic valve stenosis, *AVI* aortic valve insufficiency, *MVS* mitral valve stenosis, *MVI* mitral valve insufficiency, *CKD* chronic kidney disease, *eGFR* estimated glomerular filtration rate, *CAD* coronary artery disease, *ACS* acute coronary syndromes

In the group of deceased patients, assessment of the correlation between eGFR and the average survival time calculated by Cockcroft-Gault formula have shown a weak, positive correlation (*R* = 0.122, *p* = 0.048) (Table 4).

**Table 4:** Correlation between eGFR and mortality among patients with and without CKD

	eGFR using MDRD ml/min	GFR using Cockcrofta-Gaulta ml/min	eGFR using CKD-EPI ml/min/1.73 m^2^
Patients with CKD			
eGFR	48.71 (SD = 15.3)	47.86 (SD = 17.7)	46.05 (SD = 14.8)
Survival days	1354.8 (SD = 1136)	1354.8 (SD = 1136)	1354.8 (SD = 1136)
*R* score	0.080	0.12	0.097
Statistical significance	*p* = 0.20	*p* = 0.048	*p* = 0.12
Patients without CKD			
eGFR	87.8 (SD = 15.0)	82.62 (SD = 24.6)	77.1 (SD = 11.6)
Survival days	1536.9 (SD = 1469)	1536.9 (SD = 1469)	1536.9 (SD = 1469)
*R* score	− 0.01	− 0.06	− 0.06
Statistical significance	*p* = 0.12	*p* = 0.35	*p* = 0.29

*eGFR* estimated glomerular filtration rate, *CKD* chronic kidney disease

Increased risk of mortality was associated mostly with age (OR: 1.02, 95%CI: 1.00–1.03, *p* < 0.001), creatinine (OR: 1.27, 95%CI: 1.12–1.43, *p* < 0.001), CKD (OR: 1.30, 95%CI: 1.17–1.44, *p* < 0.001), reduced ejection fraction (EF) (OR: 0.98, 95%CI: 0.97–0.99, *p* = 0.01) and aortic valve stenosis (OR: 1.19, 95%CI: 1.04–1.35, *p* = 0.01). The factor that improved prognosis was cardiac surgery of heart valve defects (OR: 0.87, 95%CI: 0.79–0.96, *p* < 0.001) (Table 5).

**Table 5:** Wald test backward stepwise regression analysis—significant risk factor for mortality (Nagelkerke *R*^2^ = 0.49; *p* < 0.001)

	RR	95% CI	*p*
Age (years)	1.02	1.00–1.03	*p* < 0.001
Valve surgery	0.87	0.79–0.96	*p* < 0.001
Creatine (mg/dl)	1.27	1.12–1.43	*p* < 0.001
CKD	1.30	1.17–1.44	*p* < 0.001
EF (%)	0.98	0.97–0.99	*p* = 0.01
Aortic valve stenosis	1.19	1.04–1.35	*p* = 0.01

*CKD* chronic kidney disease, *EF* ejection fraction

## Discussion

Valvular heart diseases, in particular aortic stenosis, are a widespread problem for modern medicine. In the Euro Heart Survey on Valvular Heart Disease registry, aortic valve stenosis accounted for 34% of cases. It is the most common heart valve defect among European population and requires the greatest number of interventions (46.6%). Mitral valve regurgitation occurred in 25% of patients [4]. According to the WadPol Registry of Heart Diseases, the largest Polish registry of valvular diseases, the most prevalent defects were mitral regurgitation (38%) and aortic valve stenosis (27.5%). The analysis of the valvular disease etiology revealed the dominance of degenerative over rheumatoid etiology of aortic valve diseases, 50.2% vs. 30.6% [12]. Mitral valve disease presented a similar etiology pattern, with a degenerative background in 41.2% of patients, and rheumatoid etiology in every third patient (30.7%). Patients with aortic stenosis and mitral regurgitation were the oldest in "WadPol" (both groups on average 67 years old). Similar result was achieved in Euro Heart Survey (69.1 and 65.1 years, respectively) [3, 12].

Chronic kidney disease affected 37.1% of analyzed group and it was higher percentage than in other population studies, for example NHANES (14.2%) [13]. Population with chronic kidney disease is significantly older, mostly male gender and more often combined with comorbidities such as: chronic coronary syndromes (CCS), atrial fibrillation (AF) and CAD. It is well known that physiologically glomerular filtration rate decreases with age, kidney function deteriorates, for example, excretion of some ions and metabolites such as uric acid decreases [14]. Arterial hypertension and coronary artery disease, as a complication often coexists with CKD—both conditions have an additional impact on the disease [15, 16]. This leads to the progression of heart valve defects, especially those left-sided. Some studies indicate that CKD is associated with worse echocardiographic characteristics and clinical outcomes, therefore there is a need for better management of valve disease in CKD patients [17].

Samad et al. observed that patients with aortic or mitral regurgitation and coexisting chronic kidney disease have a lower survival rate and they die from cardiovascular, not renal causes [17]. Our study shows that CKD affects mostly population with mitral valve defects and numerous concomitant diseases with their complications. Causes of mitral valve defects, such as rheumatic fever in mitral stenosis or degenerative processes in mitral insufficiency are well known [18, 19]. There is a possibility that with age there is such a significant decrease in the GFR that it induces heart valve defects and other comorbidities. This conclusion can be supported by the mentioned earlier significant differences that arise with age—a gradual decline in kidney function which causes a gradual, initially asymptomatic development of complications, such as atherosclerosis and calcification which results in valve defects. Those speculations can also be endorsed by eGFR results—the analysis clearly shows that the frequency of mitral valve defects—both, severe and moderate—mostly occur with lower than 90 ml/min/1.73 m^2^ eGFR values. However, regardless of the type of valvular defect, according to the results of our study, the CKD increases the risk of death in population with heart valve defects, which strongly supports the theory that prevention and effective treatment should be performed on each stage of the disease [20].

It is worth reminding how CAD associated with significant atherosclerotic lesions and general calcification impacts on the left ventricle geometry. Chronic cardiac wall ischemia leads to worsening of cardiac function, which results in abnormal work, increased preload, ventricular remodeling and heart failure in the end. Ventricular remodeling, especially eccentric hypertrophy induces heart valve defects such as mitral insufficiency and less often aortic insufficiency [21]. The general calcification process affects not only arteries, but also heart valves, which leads to aortic and mitral stenosis [22]. Aortic stenosis and aortic insufficiency are defects mainly associated with risk factors such as age and correlated with its general calcification, degeneration and inflammatory processes. However our study shows that population with this diseases is significantly younger than those with mitral valve defects. Some studies suggest that aortic valve calcification might be an early indicator of coronary artery disease [6, 22–26]. However, this only applies to the population without CKD. Male gender in advanced age independently of CKD is more exposed to aortic valve and coronary arteries calcification [6, 22]. Our research confirms this theory—population without chronic kidney disease, more often suffering from aortic valve diseases were males. It is undeniable that CKD has a significant impact on valvular defects. It affects the prognosis of people with these defects and has an impact on their course but it is not the main initiator of aortic stenosis and insufficiency [9].

Literature reports about aortic stenosis influence on mortality among dialyzed patients with CKD [27]. According to our study, the average observation time of dialyzed population was 953 days. Among this group there were three patients with aortic stenosis and four with mitral insufficiency. According to the Euro Heart Survey and WAD-POL, the most common heart valve defects were associated with dialysis, especially mitral insufficiency, which is also the most widespread defect among population from our study.

Due to the progressive character of the heart valve disease, there is no effective prophylaxis, early treatment and diagnosis, because of lack of characteristic and in most cases—any symptoms. Secondary and primary prevention of hyperuricemia can slow the progression of CKD. Uric acid is an independent initiating CKD factor [28–30]. Maintaining a normal level of uric acid protects against further development of CKD, which is also a method of prevention against valvular defects, such as mitral regurgitation, which is the defect with the highest mortality rate, occurring mainly in the population with end-stage renal disease. Moreover, hyperuricemia was largely observed among the deceased people. Fibrinogen can also be used for this purpose in patients with and without CKD. It is quite a nonspecific marker because its growth can herald cardiovascular or non- cardiovascular mortality [31]. However, as our analysis shows, higher levels of fibrinogen were observed in the CKD population, which dominated overall mortality. Furthermore, some authors also claim that elevated fibrinogen levels can be used as a mortality predictor in the CKD population [32]. To use fibrinogen and hyperuricemia as survival markers, more detailed, long-term studies should be performed on a greater research group. Lower left ventricular ejection fraction (EF) is another factor that increases the risk of death. This condition is mainly associated with coronary artery disease and heart failure [33, 34]. Thus early prophylaxis and proper CAD treatment can significantly decrease death hazard. As far as pharmacological treatment, ACEI and statins are recommended due to their pleiotropic effects [35]. However, reduced EF has a multifactorial background [34]. Mentioned earlier aortic stenosis, which is one of the greatest death risk indicators also leads to this condition [36, 37]. Therefore, detailed diagnosis is a key tool in identifying reduced EF etiology.

Currently, there are minimally invasive methods for the correction of valvular defects, especially aortic stenosis. They are dedicated for patients with high risk for surgery [38–41]. Our study shows that cardiac surgery and percutaneous intervention of valvular defects significantly improved prognosis and reduced mortality, which was also confirmed by other authors [42, 43]. However, it should be remembered that eligibility for invasive correction of defects requires certain criteria to be met [4]. Regular echocardiography observation should be performed to control the heart valve and cardiac condition. With each modification in the echocardiographic image, the presence of new symptoms can significantly change the method of treatment [4]. As our analysis confirms, early echocardiography changes recognition can improve the comfort of patients' life and also their prognosis.

The comparison of dead and living patients showed that higher mortality occurred in the group of patients with severe mitral regurgitation, 62.9% vs. 37.1%. Deceased patients more often had significant chronic and acute coronary syndromes and CKD which was confirmed as the factor that increases the risk of mortality. The independent mortality risk factors were age and aortic regurgitation. In the current literature, mortality risk factors in patients with valvular heart disease are: age, type and stage of progression of the valvular defect, low ejection fraction of the left ventricle, coexistence of coronary artery disease, high NYHA class, hypertension, diabetes, chronic kidney disease and pulmonary hypertension [44]. According to the results, CKD is a major problem of an ageing population. It can be associated with numerous comorbidities which lead to this disease. Therefore, early diagnosis of CKD is essential as well as implementation of appropriate treatment to improve survival among high—risk patients.

### Limitations

It was a single-center-based study, it may not have general implications but the strength of this study was a large group of patients and a long follow-up period.

## Conclusions

Mitral valve defects more often than aortic valve defects coexist with chronic kidney disease. Regardless of the stage, chronic kidney disease is an additional factor affecting the prognosis in patients with heart defects. The eGFR estimated by Cockcroft-Gault correlates more closely with prognosis compared to other methods of eGFR estimation in patients with valve heart defects.

Heart valve defect with the worst prognosis is severe MVI, which may be due to the higher incidence of chronic kidney disease in this group. Other factors increasing the risk of death were age, creatinine concentration, aortic stenosis co-existence and reduced left ventricular systolic fraction. Cardiac surgery of the valve defect improved prognosis.

The monitoring of renal function in patients with VHD should be crucial as well as the implementation of treatment at an early stage.
